# Early onset chronic arthritis associated with chromosome deletion 22 q11.2 syndrome. Is it juvenile idiophatic arthritis?

**DOI:** 10.1186/1546-0096-9-S1-P167

**Published:** 2011-09-14

**Authors:** Esmeralda Nuñez, Pablo De Cabo, Gisela Diaz, Rocio Galindo, Antonio Urda

**Affiliations:** 1 Carlos Haya Children Hospital, Malaga, Spain

## Background

The chromosome 22 q11.2 deletion causes CATH 22 syndrome, a multisistemic disease with variable phenotype and a spectrum of clinical disorders: facial malformations, cardiac outflow tract abnormalities, hypocalcemia, immune deficiency,… The chronic arthritis is rare in these children, being published around 17 cases. ItÂ´s characterized to be early-onset (under 6 years), polyarticular moderate-severe outcome and it lacks rheumatoid factor.

## Case report

We report the case of a 9 months-old infant who at the age of 5 months showed stiffness and impossibility to extend knees and elbows and swelling of the small joints in hands and feet. Somatic growth stagnation and irritability is referred by parents. Perinatal history highlights only polyhydramnios since gestation week 20. Previously he was evaluated by pediatric traumatology putting away arthrogryposis. Clinical examination revealed facial abnormalities with low implantation ears, hypertelorism and inflammatory synovitis of many joints, including knees and seven small joints of hands and feet. FISH analysis exposed a deletion at the 22q11 locus. Other results are summarized in Table 1. He was initially treated with intra-articular steroids in knees and NSAIDs with partial response. Treatment with subcutaneous methotrexate and oral steroids were initiated with sustained improvement at the age of 11 months. At the same time we placed long repositioning splints (KAFO, Knee Ancle Foot Orthesis) during night and he received an intensive ambulatory physiotherapy program. After 5 months of treatment he presented knee completed extension, walks without support and the swelling in hand and feet fingers is lower allowing corticosteroids removal. Currently he is not showing any side effects or functional sequelae because of treatment.

**Table 1:** Clinical manifestations and laboratory characteristics

Cardiac abnormality	NO
**Thymus**	Not seen in ultrasound

**Inmunodeficency**	Ig G/A/M:615/64/60 mgr/dl CD4:799(27,4%) CD4/CD8:1,32

**Hypocalcemia**	NO

**ANA/RF/uveitis**	Negative/negative/No

**Dysmorphic features**	Ears low-set Hypertelorism

**Growth**	Height < p3 Weight < p3

**Neurologic development**	Normal but slow language development

**Figure1:**
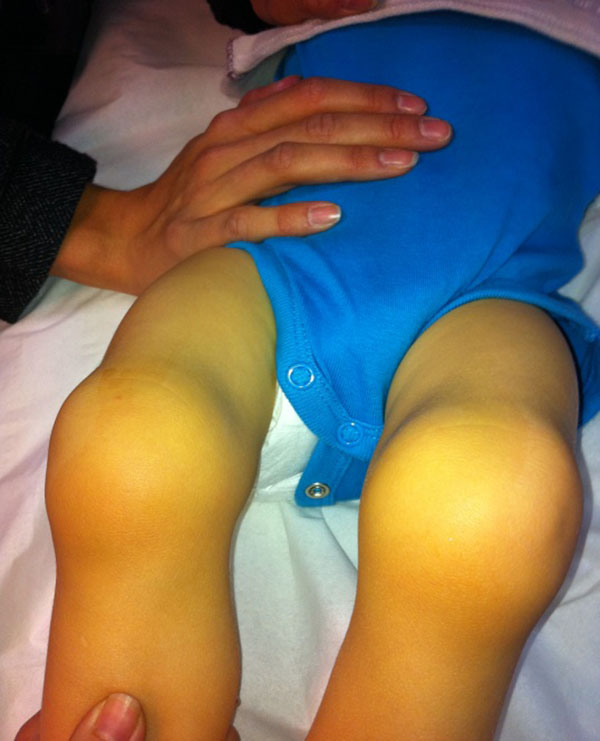
Sweeling knees in CATH 22 patient

## Conclusions

The articular disease is possible in CATCH 22 diagnosed patients, therefore we consider a follow up is needed in order to avoid long-term sequelae. In spite of the increased susceptibility to infection of these children, sometimes immunosuppressive treatment for the arthritis is needed.The use of passive nocturnal orthosis in the inflammatory phase along with a monitored physiotherapy and stimulation program helps to improve the functional abilities.

